# Family members' satisfaction with care and decision‐making in intensive care units and post‐stay follow‐up needs—a cross‐sectional survey study

**DOI:** 10.1002/nop2.97

**Published:** 2017-10-18

**Authors:** Gro Frivold, Åshild Slettebø, Daren K Heyland, Bjørg Dale

**Affiliations:** ^1^ University of Agder Faculty of Health and Sport Sciences Grimstad Norway; ^2^ Clinical Evaluation Research Unit Kingston General Hospital Kingston ON Canada; ^3^ The Canadian Researchers at the End of Life Network Kingston ON Canada; ^4^ Critical Care Nutrition Department of Critical Care Medicine Queen's University Kingston ON Canada; ^5^ Centre for Caring Research Southern Norway Grimstad Norway

**Keywords:** communication, decision‐making, family satisfaction, follow‐up, intensive care, survey study

## Abstract

**Aim:**

The aim of this study was to explore family members' satisfaction with care and decision‐making during the intensive care units stay and their follow‐up needs after the patient's discharge or death.

**Design:**

A cross‐sectional survey study was conducted.

**Methods:**

Family members of patients recently treated in an ICU were participating. The questionnaire contented of background variables, the instrument Family Satisfaction in ICU (FS‐ICU 24) and questions about follow‐up needs. Descriptive and non‐parametric statistics and a multiple linear regression were used in the analysis.

**Results:**

A total of 123 (47%) relatives returned the questionnaire. Satisfaction with care was higher scored than satisfaction with decision‐making. Follow‐ up needs after the ICU stay was reported by 19 (17%) of the participants. Gender and length of the ICU stay were shown as factors identified to predict follow‐up needs.

## 1 INTRODUCTION

Admission of critically ill patients to intensive care units (ICUs) usually includes involvement of close family members. Family members are essential to the patient both during and after the hospital stay for promoting the patient's psychological well‐being and enhancing the patient's motivation to remain alive and fight back against illness (Bailey, Sabbagh, Loiselle, Boileau, & McVey, 2010; Engström, Uusitalo, & Engström, 2011). However, ICU experiences affect family members in many ways, which necessitates special attention to the family members' own needs and reactions (Fumis, Ranzani, Faria, & Schettino, 2014).

Family members of ICU patients are a vulnerable group with a high risk of decline in their own health (Baumhover & May, 2013). This may be due to the uncertainty and fear associated with the patients' critical illness and the frightening impressions associated with the ICU environment (Henrich et al., 2011; van Mol et al., 2014).

According to the principle of patients' autonomy, healthcare decisions involving serious interventions should be based on informed consent (Patients' Rights Act, 2016). However, because ICU patients often lack decision‐making capacity, family members frequently must act as surrogate decision‐makers (Huffines et al., 2013). When families and health‐care professionals engage in this process as equals, this is called shared decision‐making (Barry & Edgman‐Levitan, 2012). Meaningful participation in such decision‐making processes requires that participating family members have sufficient information to weigh the potential benefits and burdens of treatment and that they have obtained understanding of likely outcomes for the patient (Azoulay, Kentish‐Barnes, & Nelson,^2016^). Furthermore, Barry and Edgman‐Levitan (2012) claim that a good decision is one that best aligns a patients' values and preferences with the treatment that is most likely to result in an outcome wanted by the patient. In Norway, contrary to the practises in the North American countries, family members legally have limited decision‐making power and the final decision‐making responsibility is left to the physician (Moselli, Debernardi & Piovano, 2006; Patients' Rights Act, 2016). Despite various practices, experiences with the decision‐making process often contribute further stress in an already challenging time, which is full of shock and confusion. In this article, decision‐making is used both related to information exchange between the patient, the family members and the health‐care providers about basic treatment options and major decisions related to end of life care. To decrease the psychological burden, care of family members during the ICU stay has been shown to be crucial (Azoulay et al., 2005; Schmidt & Azoulay, 2012). This requires, among other aspects, high‐quality communication between family and health‐care staff (Heyland, Rocker, O'Callaghan, Dodek, & Cook, 2003; Huffines et al., 2013; Kryworuchko & Heyland, 2009).

To improve the quality of care provided to families of patients in the ICU, assessing family members' satisfaction with the care and support they receive is important (Kryworuchko & Heyland, 2009). Although studies have shown that family members are generally highly satisfied, (Heyland et al., 2002; Stricker et al., 2009), several areas for improvement have been identified, such as communication with physicians, involvement in patient care and decision‐making (Dodek, Heyland, Rocker, & Cook, 2004) and the atmosphere in the ICU waiting room (Osborn et al., 2012). Several studies have focused on measuring family satisfaction at patients' end of life (Wall, Engelberg, Downey, Heyland, & Curtis, 2007). However, it also is important to consider families of survivors because most ICU patients survive (Hunziker et al., 2012; Iwashyna, 2010). Surviving from critical illness will affect the lives of both the survivors and their family members (Bremer, Dahlberg, & Sandman 2009; Israelsson, 2016).

Based on the growing knowledge of major health consequences in patients and family members after an ICU stay (Rosendahl, Brunkhorst, Jaenichen, & Strauss, 2013; Wallin, Larsson, Rubertsson, & Kristoferzon, 2013), there is an increasing focus on the importance of post‐ICU follow‐up, mainly addressing the perspective of the patients (Egerod et al., 2013). Intensive care follow‐up programmes, mainly led by ICU nurses, have emerged over the past decade (Svenningsen, Langhorn, Agard, & Dreyer, 2017). Such programmes are usually described as hospital re‐visits and dialogue between the patient and health‐care professionals (Egerod et al., 2013), which have been positively evaluated (Egerod et al., 2013; Engström et al., 2008; Zoellner & Maercker, 2006). However, there is an insufficient number of studies that focus on family members' follow‐up needs. Regardless of the patient's outcome, impressions and experiences from an ICU stay may also affect family members after the discharge or death of the patient, as these family members can manifest symptoms of anxiety and depression that often tend to persist over time (Azoulay et al., 2005).

Furthermore, to our knowledge, no study has focused on the relationship between satisfaction with the hospital stay and the need for ICU follow‐up after discharge or death of the patient.

In this paper, the term − family members − is used and refers to the person closely involved in the process of patient's ICU admission and stay. This might have been the spouse, children or parents, but could also be friends or other people close to the patient (Friedman, Bowden, & Jones 2003).

The aim of this study were:

1. To explore family members' satisfaction with care and decision‐making during the ICU stay
2. To explore their follow‐up needs after the patient's discharge or death
3. To explore possible associations between family members ‘satisfaction with care and decision‐making during the ICU stay and follow‐up needs.

## 2 THE STUDY

### 2.1 Design

This study was a cross‐sectional survey performed among close family members of former ICU patients.

### 2.2 Participants and setting

Fifteen ICUs in the south of Norway were invited to participate and nine ICUs accepted. These ICUs were at local, regional or university hospitals. Most ICUs treated both medical and surgical patients. The inclusion criteria were being a close family member of an intensive care patient who was ≥18 years of age and who stayed in the ICU for at least for 24 hr. The family member group (participants) also had to be ≥18 years of age. Participants were limited to those family members who were within the range of 2 months to 1 year's time since the patient was discharged from ICU (or death) and the participants had to be able to understand and express themselves in the Norwegian language. Family members, one per patient, were identified from the hospitals' electronic register of intensive care patients.

The questionnaire along with written information about the study and participants' rights was mailed to 261 eligible family members. Returned questionnaires presumed participation consent. Demographic data on the non‐participants in the study (N = 143) were not collected. Due to research ethics, we chose not to approach these persons to investigate their reasons for not participating.

### 2.3 Data collection

The questionnaire consisted of demographic questions, one validated instrument and questions regarding the family members' life after the ICU stay and the family members' follow‐up needs. The demographic questions included are presented in Table 1. The 24‐item version of the Family Satisfaction in the Intensive Care Unit (FS‐ICU 24), developed by Heyland and Tranmer (2001), was used for measuring family satisfaction with ICU care and decision‐making. The FS‐ICU 24 provides a total satisfaction score (FS‐Total) for 24 items and two subscales that rate satisfaction with care (FS‐Care), 14 items and with decision‐making (FS‐DM), 10 items. The FS‐Care subscale includes questions about care of the patient and family members as well as nurses' communication skills. The FS‐DM subscale includes questions addressing communication with physicians about the condition of the patient, sufficient time for decision‐making and quality of information and involvement in the decision‐making process. Each item, except one that is dichotomous, (question 21), was scored on a 5‐point Likert scale with the following options: (i) poor; (ii) fair; (iii) good; (iv) very good; (v) excellent. The scale was recoded, per the recommended procedure (Wall et al., 2007), into scores ranging from 0‐100, (0 = poor, 25 = fair, 50 = good, 75 = very good and 100 = excellent). Transforming item scores to a 0–100 scale was an important step that makes the values more meaningful and more appropriate for statistical analyses. As described by Wall et al. (2007), scores are calculated by averaging the available items, provided the respondents answered at least 70% of the respective items. The FS‐ICU 24 has been found to be reliable and valid in former studies (Rothen, Stricker, & Heyland, 2010; van den Broek et al., 2015). The instrument has been translated into several languages (Rothen, Stricker, & Heyland, 2010; Wall et al., 2007) but not into Norwegian at the time of this survey. Hence, the authors obtained permission to translate the questionnaire FS‐ICU‐24 for use in Norwegian contexts. The original version of the English language questionnaire (Heyland & Tranmer, 2001) was translated into Norwegian by a bilingual professional translator, in accordance with the translating and back‐translating procedure recommended by Streiner and Norman (2008). The back‐translated version of the questionnaire was reviewed by the constructor of the instrument to ensure that the original meaning of the included items was maintained.

**Table 1:** Characteristics of patients and participants

Patients (N = 123)		
Age in years, *M* (*SD*)	68	(15)
Females, *n* (%)	46	(37.4)
Died during ICU stay, *n* (%)	40	(32.5)
Weeks since the ICU stay, mean (*SD*)	30	(14.4)
Length in days of ICU stay, mean (*SD*)	19	(24.0)
Diagnosis, *n* (%)		
Heart disease	45	(36.6)
Trauma	10	(8.1)
Respiratory failure	27	(22.0)
Abdominal disease	27	(22.0)
Neurological disease	4	(3.3)
Others	37	(30.0)
Mechanically ventilated, *n* (%)	80	(65.0)
Participants (N = 123)		
Age in years, *M* (*SD*)	60	(13)
Females, *n* (%)	73	(59)
Education level, *n* (%)		
Elementary school	20	(16.3)
Secondary school	48	(39.0)
University college/university	49	(39.8)
Relationship to patient *n* (%)		
Spouse	64	(52.0)
Parents	14	(11.4)
Children	33	(26.8)
Siblings	6	(4.9)
Others	3	(2.4)

In addition to this instrument, the questionnaire consisted of questions developed by the authors, regarding follow‐up needs in family members after the ICU stay. They were asked if they had follow‐up needs with the options: “not at all”, “slightly”, “moderately”, “largely” or “very great extent”. In addition, family members were asked whether they had been followed up by the ICU (“yes” or “no”) and who initiated the follow‐up (“I contacted the ICU”, “the ICU contacted me” or “others contacted the ICU on my behalf”). The type of follow‐up was queried (“telephone call”, “follow‐up re‐visit in the ICU”, “follow‐up re‐visit with the patient”, “other follow‐up activities”). To what extent these interventions were helpful was assessed by the same 5‐point Likert scale options (ranging from “not at all”, to “very great extent”). At the end of the questionnaire, participants were asked to express in their own words their thoughts about the ideal follow‐up after the ICU stay.

The questionnaire, including the translated version of the instrument, was reviewed by five intensive care professionals in a Norwegian ICU and a pilot study was conducted among six close family members of patients who had recently stayed in an ICU. Both the professionals and the family members were asked to respond to and evaluate the translated version of the questionnaire. They found the questions easy to understand and meaningful for measuring family satisfaction in ICUs. The size and the structure of the questionnaire were also considered to be appropriate. Minor changes were made in the questionnaire after the pilot study.

### 2.4 Data analysis

Data analysis was performed using the IBM SPSS Statistics for Windows Version 22.0 (Armonk, NY, USA, IBM Corp). The reliability of the Norwegian version of FS‐ICU 24 was assessed by estimating the internal consistency (homogeneity) with item‐to‐total correlations, reflecting to what degree all items in the instrument measure the same attribute. Internal consistency was calculated by Spearman's rank correlations (*r*_s_) between each item and the total instrument, after omitting the individual item from the total instrument. Internal consistency was also estimated with Cronbach's alpha reliability coefficients for the FS‐Total and the FS‐Care and FS‐DM subscales.

Descriptive analyses were generated for patients' and family members' demographics and for the scores on each FS‐ICU 24 item, total scales and sub scales of the FS‐ICU 24. Differences in the satisfaction scores according to groups of family members were calculated by use of non‐parametric statistics, that is, the Mann–Whitney U‐test and the Kruskal–Wallis test, due to the non‐normally distributed data. The same analyses were performed to explore possible differences in follow‐up needs among the same groups of family members.

Spearman's correlation analysis was performed to identify potential associations between the 24 single items in the FS‐ICU 24 and follow‐up needs. A multiple, linear regression analysis was performed with the dependent variable “need of follow‐up”. Based on results from the univariate analyses for investigating significant relationships (*p* < .05), the following independent variables were included in the regression analysis: gender family members, length of stay in the ICU, care of patient, management of breathlessness, staff providing understandable explanations and the staff's willingness to give information. In examining the variation of inflation factors in the model, no consequential multi‐collinearity between the independent variables was found. The model was also tested for outliers, normality, linearity, homoscedasticity and independence of residuals. Respondents with pairwise missing values (N = 27) were excluded from the analyses.

The participants' answers to the open‐ended questions about reasons for having/not having follow‐up needs and the ideal follow‐up after the ICU stay were categorized and labelled in a way that the most frequently occurring themes in the written text were summarized and labelled as text categories.

### 2.5 Ethics

Prior to data collection, the study was approved by the Regional Committee for Medical Research Ethics in Norway (Reference number: REK Sørøst A 2013/458) and from each participating ICU.

## 3 RESULTS

A total of 123 family members were included in the study. The response rate from family members was 47, 1%. Forty family members (32.5%) were of non‐survivors and 83 (67.5%) were survivors' family members. Table 1 displays the demographics of the participants and the patients.

Cronbach's alpha coefficient for the FS‐Total scale was 0.96 and for the subscales FS‐Care and FS‐DM, 0.93 and 0.94 respectively. The results indicate a high level of homogeneity of the instrument. The homogeneity was also reflected in the item‐to‐total correlation analyses as all items were significantly correlated to the total instrument with a *p*‐value of *p* < 0.01. Twenty‐one of the items reached a correlation coefficient of *r*_s_ ≥ .60. The three items with lower correlation coefficients to the total instrument were “Skill and competence of nurses” (*r*_s_ = .56), “Atmosphere of the ICU waiting room” (*r*_s_ = .45) and “Satisfaction of the level or amount of care the patient received” (*r*_s_ = .35).

### 3.1 Satisfaction with care and decision‐making during the ICU stay

The family members were satisfied with the treatment and care for the patient, but less satisfied with the care of the family members and the ICU staffs' communication skills (Table 2). The subscale FS‐care showed a mean score of 73 of 100 (*SD* 20.2) and the subscale FS‐decision‐making showed a mean score of 64.8 (*SD* 26.3). Total score, FS‐total showed a mean of 70.0 (21.7). The scores of the single items on the FS‐ICU 24 are presented in Table 2.

**Table 2:** FS‐ICU 24 single item scores, mean (*M*) and standard deviation (*SD*)

Satisfaction with care and decision‐making in the ICU	N	Exellent (%)	Very good (%)	Good (%)	Fair (%)	Poor (%)	*M*	(*SD*)
1. Courtesy, respect and compassion by staff toward patient	122	48.4	37.7	8.2	4.9	0.8	82.0	(21.9)
2. Management of pain	117	43.6	37.6	14.5	2.6	1.7	79.7	(22.5)
3. Management of breathlessness	104	43.3	40.5	13.5	1.9	1.0	80.8	(20.7)
4. Management of agitation	105	34.3	45.7	11.4	6.7	1.9	76.0	(23.7)
5. Considering family needs	120	33.3	36.7	15.8	7.5	6.7	70.6	(29.3)
6. Emotional support towards family	114	29.8	31.6	20.2	12.3	6.1	66.7	(30.1)
7. Coordination and teamwork by staff	118	32.2	41.5	19.5	5.1	1.7	74.4	(23.4)
8. Courtesy, respect and compassion by staff toward family	121	37.0	39.7	14.9	5.8	1.5	75.8	(24.8)
9. Skills and competence of nurses	123	45.5	43.9	7.3	3.3	0.0	82.9	(18.6)
10. Communication by nurses	121	32.2	30.6	18.2	9.1	9.9	66.5	(32.2)
11. Skill and competence of doctors	118	40.7	31.4	19.5	4.2	4.2	75.0	(27.0)
12. Atmosphere of the ICU	106	34.4	40.2	16.4	5.7	3.3	74.2	(25.5)
13. Atmosphere of the waiting room	122	12.3	23.6	31.1	22.6	10.4	51.2	(29.4)
14. Satisfaction of the level or amount of care patient received	119	21.0	34.5	16.0	5.9	5.9	62.2	(29.1)
15. Frequency of communication by doctors	114	18.4	28.9	20.2	10.5	21.9	52.9	(35.5)
16. Willingness of staff to answer questions	121	33.9	38.0	9.9	11.6	6.6	70.2	(30.3)
17. Staff provided understandable explanations	121	29.8	33.1	17.4	15.7	4.1	67.1	(29.4)
18. Honesty of information provided about patient's condition	120	34.2	34.2	19.2	5.0	7.5	70.6	(29.5)
19. Completeness of information about what was happening	121	31.4	31.4	17.4	7.4	12.4	64.5	(33.3)
20. Consistency of information about patient's condition	114	26.3	34.2	24.6	6.1	8.8	65.8	(29.8)
21. Enough time in decision‐making (dichotomous)	107	71.0				29.0	71.0	(45.6)
22. Feeling control in decision‐making	118	25.4	16.1	44.1	7.6	7.6	61.1	(29.2)
23. Feeling supported in decision‐making	115	18.3	30.4	38.3	4.3	4.3	62.4	(25.7)
24. Feeling included in decision‐making	118	21.2	26.3	43.2	6.8	6.8	63.1	(26.7)

Family members of patients who died during the stay were more satisfied with support in decision‐making compared with family members of survivors (mean = 72.4 vs. 59.7, *p* = .01) and with inclusion in decision‐making (mean = 73.3 vs. 60.1, *p* = .01). Family members of patients who were mechanically ventilated in the ICU were more satisfied with care and decision‐making compared with family members of patients who were not mechanically ventilated (FS‐Total: mean = 74.8 vs. 58.0, *p* = .01; FS‐Care: mean = 77.2 vs. 62.6, *p* = .01; FS‐DM: mean = 70.9 vs. 51.8, *p* = .002).

### 3.2 Family members' satisfaction with and needs for follow‐up after the ICU stay

A total of 96 participants (83%) reported that they had no or very limited follow‐up needs. Length of stay in the ICU and gender of family members was found as predictors for follow‐up needs. An increased length of ICU stay was associated with greater follow‐up needs and gender predicted greater follow‐up needs. The explained variance, Beta and p‐values of the included items are shown in Table 3.

**Table 3:** Predictors for relatives' follow‐up needs: multiple, linear regression analysis

*R* ^2^ = .161	Unstandarized	Explained variance	*p*‐value
	Beta	(%)	
Gender relatives	0.38	0.05	.041
Length of stay	0.01	0.08	.005
FS Patient care	−0.09	0.00	.595
FS breath	0.14	0.00	.447
FS quest	0.13	0.00	.359
FS expl	0.19	0.01	.172

FS Patient care = Family satisfaction with care of the patient

FS breath = Family satisfaction with management of patient's breathlessness

FS quest = Family satisfaction with staff providing understandable explanations

FS expl = Family satisfaction with staff's willingness to give information

Those who had experienced some sort of follow‐up (N = 19) reported that it consisted mostly of telephone conversations (52%) and accompanying the patient to follow‐up meetings (30%). These follow‐up activities were reported as being helpful by 40% of family members who had such experiences. Non‐survivors' family members received more follow‐up than survivors' family members (*p* < 0.01).

Nineteen family members (17%) reported follow‐up needs. Only two of them received a follow‐up from the ICU. The family members mentioned several reasons when they were asked to express their thoughts about follow‐up needs (Table 3). Some had experienced a high quality of care and information during the stay and did not have further follow‐up needs after the stay. Others had suffered from a lack of comprehensibility and needed clarification related to information or additional information. There were family members who reported a lack of trust and confidence in the ICU staff because of the ICU experience and who did not want further contact. There could also be a need for practical help and support that was not connected to the satisfaction with the ICU stay. The participants in the present study also had suggestions for the ideal follow‐up after the ICU stay, such as being contacted after a while at home, having a contact person to call in the ICU or being invited to a revisit to have the opportunity to ask questions or show their appreciation. Reasons related to follow‐up needs and suggestions for follow‐up interventions are presented in Table 4.

**Table 4:** Reasons related to follow‐up needs in relatives

Reasons for not having follow‐up needs	Reasons for having follow‐up needs	Suggestions for the ideal follow‐up after the ICU stay
Good information and care during the ICU stay Personal strength Support from close family, friends or family doctor. Lack of confidence in the ICU because of negative feelings	Lack of information and comprehensibility Lack of contact and communication with doctors during the stay Need for someone to talk to about what happened Need for personal care, practical help, support Need for help related to personal feelings and reactions	Telephone call after a few weeks to clarify information Having a telephone number available when needing to contact the ICU Follow‐up meeting in the ICU Professional support after the ICU stay related to relatives' own emotional reactions

## 4 DISCUSSION

Most family members in the present study were satisfied with the care of the patient and the skills and competence of nurses and physicians. Satisfaction with communication and care of the family members was lower. Most family members reported limited needs for follow‐up, but females and those with family members having a longer stay in ICU reported higher needs.

The results showing that the family members were more satisfied with care provided to the patient than with the care they received themselves are in accordance with other studies (Carlson, Spain, Muhtadie, McDade‐Montez, & Macia, 2015; Fumis, Nishimoto, & Deheinzelin, 2008; Heyland et al., 2002; Sundararajan, Sullivan, & Chapman, 2012). Reasons for this pattern, as suggested by Carlson et al. (2015), relate to the emotional distress of the family members and the nature of the ICU staff workload that makes it unrealistic to fulfil the expectations of every family when being responsible for numerous critically ill patients. In addition, health‐care providers themselves can be distressed, which might complicate interaction with the family members (Carlson et al., 2015). Even if such explanations are recognized, family care requires communication skills in meeting the family members' needs for information and support. Since ineffective communication and inconsistency in ICU staff communication is associated with a higher risk of symptoms of depression in family members (Hwang et al., 2014; Pochard et al., 2001), all members of an ICU team should be informed of the treatment goals for any/every patient. Regular family meetings, especially before discharge from the ICU, are suggested to ensure that family members receive the information required (Stelfox et al., 2015).

Our study showed that family members of patients who were mechanically ventilated were more satisfied than other family members. Higher satisfaction with family care has been associated with higher severity of illness (Stricker et al., 2009) and, according to the “standards for nurse staffing in critical care units”, Bray et al. (2004) suggested that a higher registered‐nursing staff to patient ratio has a positive impact on the outcomes of patients and families.

Furthermore, the family members of non‐survivors were more satisfied regarding inclusion and support in the decision‐making processes. This difference in experience for the inclusion of family members can probably be explained by some patients being able to participate in the decisions themselves and the conflicts in opinion between the patient, the health‐care staff and the family members. There seems to be more focus on inclusion of family members when ICU patients are dying. End of life care has been increasingly promoted in the ICU field and several studies have investigated its value (Cabrini et al., 2016; Heyland et al., 2003; Kross et al., 2011; Wessman, Sona, & Schallom, 2015). However, all family members need to be integrated in the communication process, regardless of patient severity or outcome (Cox, White, & Abernethy, 2014). Effective communication ensuring that family members understand the nature of the patient's condition, including diagnosis, prognosis and treatment risks and benefits is crucial for family members to cope with their role as substitute decision‐makers (Azoulay et al., 2001).

Several of the family members in this study were not satisfied with the atmosphere in the waiting room. Comparable results are shown in other studies (Hagerty, Velázquez, Schmidt, & Falo, 2016; Henrich et al., 2011; Heyland et al., 2002; Osborn et al.,^2012^). Given the fact that several hours are spent by families in these waiting rooms, these low family satisfaction results should generate rapid improvements in the construction of the ICU environment.

Most family members in the current study reported little need for follow‐up after the patient's discharge or death. This is in accordance with a recent Norwegian study (Frivold, Slettebo, & Dale, 2016), that concluded family members seem to be able to cope by using their personal resources in addition to support from friends and other family members. However, family members' answers on the open‐ended questions showed that areas reflecting quality of care and decision‐making during the ICU stay were frequently mentioned when describing their follow‐up needs.

Needs for follow‐up in the study group was connected to insufficient information and care during the stay or new challenges after discharge that required further support, such as the need for someone to talk to about what happened or the need for help related to personal feelings and reactions. Family members are found to need more information related to the patient's treatment for one to 2 years after the patient returns home and require professional assistance but did not know whom to turn to (Czerwonka et al., 2015). Some family members are also responsible for the care of the patient after discharge (Agard, Egerod, Tønnesen, & Lomborg, 2015; Steenbergen et al., 2015). This could explain why some family members in the present study reported a need for follow‐up after the ICU stay even if they were generally satisfied with care during the stay.

Negative effects of an ICU stay on family members' psychological health is not limited to only bereaved family members (Anderson, Arnold, Angus, & Bryce, 2008), hence, it seems that the current practice of targeting family members of non‐surviving patients for follow‐up is insufficient.

The family members' gender was associated with follow‐up, as women reported significantly higher needs than men and gender was shown as a predictor for follow‐up needs. According to Dahlberg, Demack, and Bambra (2007), most informal caregivers are women who receive less assistance from family and friends compared with men and outsiders are more likely to assist a male caregiver rather than a female caregiver. Studies have concluded that women's health is more likely to be affected by caregiving burdens than men's health and women reported greater burden, stress, anxiety and depression (Navaie‐Waliser, Spriggs, & Feldman, 2002; Yee & Schulz, 2000). Hence, one might assume that women need more attention with respect to follow‐up from the ICU.

Specific groups of family members seem to need additional support after ICU, such as females and family members of patients who had a longer stay in the ICU. This would require a connection between the family and the ICU that continues after the discharge or death of the patient and a tighter collaboration between the hospitals and the municipality. Reasons given for follow‐up needs by family members were “Need for personal care, practical help and support” and “Need for help related to personal feelings and reactions”. These reasons exemplify needs that ideally should be met by other resources than the ICU staff, such as medical and practical support from home based nursing care or a psychologist.

The current study adds new information regarding family members' suggestions for helpful follow‐up strategies, including offering telephone calls after a few weeks, providing the opportunity to clarify information or participating in re‐visits to the ICU to help in handling problems. The nurses' unique position to provide insight and support to family members during the stay and to help them be aware of their own physical health, is important to prevent post‐ICU vulnerability (Choi, Donahoe, & Hoffman, 2016). Family member' follow‐up needs after ICU experiences, require further investigation to determine the most suitable support system.

### 4.1 Methodological limitations

Because of the nature of this study, participants had returned home for several months and their responses could be influenced by a recall bias. The assessment of satisfaction with care after such a long time would also be influenced by other aspects of life, such as complicated grief or heavy care burdens, which might disturb the initial impression.

The response rate of the current project was relatively low (47%), which decreases the generalizability of the study. Due to ethical considerations and the vulnerability of the study population, it was neither considered appropriate to use repeated reminders, nor was it ethically acceptable to ask about reasons for not participating. Therefore, information about the non‐participating family members is not available.

Based on the current knowledge concerning potential care burdens in family members (Agard et al., 2015; Steenbergen et al., 2015) and frequently reported emotional impairments in family members after ICU experiences (Fumis, Ranzani, Martins, & Schettino, 2015), it might be assumed that the persons most burdened were incapable of participating in the study. For some because of lack of time or interest, for others it might also be overwhelming to complete a questionnaire with several pages of questions if it was not considered a priority.

## 5 CONCLUSIONS

The family members in nine ICUs reported satisfaction with patient care and with the skills and competence of the ICU staff. However, family members' satisfaction with care of the family and the staffs' communication with the family members, was shown as areas with potential for improvement. This emphasizes the need for continued focus on communication skills in nurse and physician education and practical ICU training. Involvement in decision‐making, particularly among survivors' family members, proved to be an area for potential improvement.

Most family members in this study sample reported no need for follow‐up after the ICU stay. Family members of non‐surviving patients were receiving more follow‐up from the ICU staff after the ICU stay than family members of surviving patients. However, female family members and those with a longer ICU stay reported a higher need for follow‐up. This might imply support from resources or interventions other than the ICU staff. No association was found between family satisfaction with care during the ICU stay and their follow‐up needs after the stay. However, the current study shows that quality of care and communication during the hospital stay are important reasons for follow‐up needs and continuing improvements in these areas are required.

## CONFLICT OF INTEREST

The authors declare that there are no conflicts of interest.

## AUTHOR CONTRIBUTIONS

GF participated in the design of the study, the translation of the instrument FS‐ICU 24, and the statistical analysis. She performed the data collection, and was the main responsible for drafting the manuscript. ÅS participated in the design of the study, the translation of the instrument FS‐ICU 24, and the interpretation of the data. She commented on the drafted manuscript. DH developed the original instrument FS‐ICU 24 and he approved the translated Norwegian version. He contributed in the data analyses, and reviewed the manuscript. BD participated in the design of the study, the translation of the FS‐ICU 24 instrument, the data analyses, the interpretation of data and drafting of the manuscript. All authors read and approved the final manuscript.

All authors have agreed on the final version of the paper and meet at least one of the following criteria [based on those recommended by the ICMJE (http://www.icmje.org/recommendations/)]:

- substantial contributions to conception and design, acquisition of data or analysis and interpretation of data
- drafting the article or revising it critically for important intellectual content.
